# Use of Dexamethasone for Severe Fatigue in the Advanced Cancer Population: A Brief Report

**DOI:** 10.1089/pmr.2023.0050

**Published:** 2024-03-13

**Authors:** Gemma Ingham, Kat Urban, Yinyin Phyo, Jane Hunt, Penelope Tuffin, Davinia Seah

**Affiliations:** ^1^ Department of Palliative Care, Prince of Wales Hospital, Randwick, New South Wales, Australia.; ^2^ Department of Palliative Care, Lismore Base Hospital, Lismore, New South Wales, Australia.; ^3^ Centre for Improving Palliative, Aged, and Chronic Care Through Clinical Research and Translation (IMPACCT), University of Technology, Sydney, New South Wales, Australia.; ^4^ Palliative Care Program, Pharmacist WA Country Health Service, Western Australia.; ^5^ Pharmacy and Palliative Care Departments, Fiona Stanley Hospital, Perth, Western Australia.; ^6^ Palliative Care Unit, Bethesda Hospital, Perth, Western Australia.; ^7^ Supportive and Palliative Care, Sacred Heart Health Service, Sydney, New South Wales, Australia.; ^8^ St Vincent's Clinical School, University of New South Wales, Sydney, New South Wales, Australia.

**Keywords:** adverse events, dexamethasone, fatigue, palliative care

## Abstract

**Background:**

Fatigue is a common and distressing symptom for palliative care patients. Although the current literature emphasizes nonpharmacological management, dexamethasone is reportedly used in clinical practice. This study helps to characterize its use, efficacy, and adverse effects in a real-world setting.

**Objective:**

To improve the evidence base by exploring the use, efficacy, and side effect profile of dexamethasone for fatigue management.

**Methods:**

This international multisite prospective observational case series assessed the benefit and adverse effects of dexamethasone at baseline (T0) and at five to seven days postbaseline (T1). Fatigue scores were assessed using the symptom assessment scale (SAS) and visual analogue fatigue scale (VAFS). Adverse events were graded using the National Cancer Institute Common Terminology Criteria for Adverse Events (NCI-CTCAE). The related samples Wilcoxon signed-rank test was used to compare before and after scores.

**Results:**

All 18 patients (male–female, 11:7) had advanced metastatic cancer with most in the deteriorating palliative care phase (56%). The most common dose of dexamethasone was 4 mg daily orally. At T1 (*n* = 12), improvement was seen in all measures of fatigue; the median SAS scores decreased from 7 to 5.5 (*p* = 0.007), the median VAFS scores increased from 3 to 5 (*p* = 0.126), and the median NCI-CTCAE fatigue scores were reduced from 3 to 2.5 (*p* = 0.18). Dexamethasone was well tolerated; one participant experienced grade 3 delirium.

**Conclusion:**

The small number of participants recruited for this study suggests that dexamethasone is not widely used specifically for fatigue. Our results suggest an improvement in fatigue scores from T0 to T1.

## Introduction

Cancer-related fatigue is a distressing persistent subjective sense of physical, emotional, and/or cognitive tiredness or exhaustion related to cancer or cancer treatment that is not proportional to recent activity and interferes with usual functioning.^1^ Fatigue affects >80% of palliative care patients,^2^ causing significant distress and a poor quality of life. A study of the Australian population shows that it affects patients with malignant and nonmalignant diagnoses equally with the majority of patients experiencing moderate or severe distress that escalates as their function declines.^3^ Despite this, it is often under-reported by patients, and health care professionals fail to recognize its significance nor know how to manage it confidently, rendering it a very challenging symptom to manage.^4,5^

There is no consensus on the best management plan, but it is widely accepted that after completing a thorough assessment, including reviewing contributing symptoms and treating underlying reversible conditions such as anemia, hypothyroidism, and depression, a multimodal nonpharmacological approach should be prioritized. This includes education, individualized exercise programs, pacing, and cognitive behavioral therapy.^1,2,6^ Medications are generally not recommended although it is observed that the corticosteroid, dexamethasone, is used in practice.

The most recent Cochrane review acknowledges that although a single randomized controlled trial (RCT) shows dexamethasone to be superior to placebo in improving cancer-related fatigue, there is still inadequate evidence to support its use for this indication.^7^ The report also highlights that it is generally not recommended for long-term fatigue management due to its toxicity.

The University of Technology Sydney (UTS) Improving Palliative, Aged and Chronic Care through Clinical Research and Translation (IMPACCT) rapid program is a collaborative international program focused on the prospective use of pharmacological interventions commonly used in palliative care.^8^ This rapid series aimed to improve the evidence base by exploring dexamethasone's use, efficacy, and side effect profile for fatigue management.

## Methods

This international multisite prospective observational case series assessed dexamethasone's benefit and adverse effects over a 26-month period (January 2020 to March 2022). Full details of the methods of the pharmacovigilance program have been described previously for other medications^9–12^; exactly the same established methodology was used in this series. Clinicians collected data when dexamethasone was started for fatigue as part of routine clinical care by palliative care physicians.

Data were collected at two time points: baseline (T0) when the medication was commenced and again at five to seven days postbaseline (T1). Patients anonymized demographic and clinical data were collected and entered into a secure web application for building and managing online databases, Research Electronic Data Capture.^13^

Fatigue scores were assessed using the symptom assessment scale (SAS) for fatigue (0 being no distress from fatigue, 10 being the worst possible distress from fatigue)^14^ and the visual analogue fatigue scale (VAFS; 0 being the worst possible fatigue, 10 no fatigue).^15,16^ The related samples Wilcoxon signed-rank test was used to compare before and after scores. Adverse events were graded using the National Cancer Institute Common Terminology Criteria for Adverse Events (NCI-CTCAE) version 5 where higher scores indicate worse symptoms (grade 1 being mild symptoms with no intervention required; grade 3 being severe or medically significant but not immediately life threatening, requiring hospitalization or prolongation of hospitalization; grade 5 being death).^17^

Analyses were performed using SPSS software version 26.0 (IBM, Armonk, NY, USA).

The study received ethics approval from all participating institutions' human research ethics committees as a low-risk study or received ethical waivers as a quality improvement program reporting on prospectively collected clinical outcomes after the clinical decision to prescribe dexamethasone. Individual consent was, therefore, not required.

## Results

### Demographics

Data for 18 patients (male–female, 11:7) were collected at eight sites in three countries (Australia, New Zealand, and the United Kingdom). Patients had a median age of 70 years (interquartile range 61–83) and had advanced metastatic cancer with the most common type being lung cancer (39%). Most patients were in the deteriorating palliative care phase (56%), followed by the unstable phase (39%) at baseline. Most patients (89%) had an Australia-modified Karnofsky performance status of 60 or less. The median hemoglobin level was 110 g/L. Demographic data are summarized in Table 1.

**Table 1.** Patient Demographics

	***n*** (%)	Median (IQR)
Gender		
Female	7 (38.9)	
Male	11 (61.1)	
Age		70 (61–83)
Primary life limiting illness		
Advanced metastatic cancer	18 (100)	
Noncancer	0	
Type of cancer		
Lung	7 (38.9)	
Upper gastrointestinal	3 (16.7)	
Unknown primary	2 (11.1)	
Prostate	1 (5.6)	
Leukemia	1 (5.6)	
Not specified	4 (22.2)	
Palliative care phase		
Stable	1 (5.6)	
Unstable	7 (38.9)	
Deteriorating	10 (55.6)	
Terminal	0	
Australian-modified Karnofsky Performance Status Score		
100 (Normal)	0	
90 (Able to carry on normal activity	0	
80 (Normal activity with effort)	0	
70 (Cares for self; unable to carry on normal activity)	2 (11.1)	
60 (Requires occasional assistance but able to care for most needs)	7 (38.9)	
50 (Requires considerable assistance and frequent medical care)	3 (16.7)	
40 (In bed >50% of the time)	4 (22.2)	
30 (Almost completely bedfast)	0	
20 (Totally bedfast and requiring extensive nursing care)	1 (5.6)	
Not able to determine	1 (5.6)	
Charlson comorbidity index		5.5 (3–10)
Hemoglobin (*n* = 14)		110 g/L (98–128)

IQR, interquartile range.

### Dose and route of administration

Table 2 summarizes the dose and administration route of dexamethasone at baseline. At T0 (*n* = 18), the majority were given a daily morning (94%) dose of dexamethasone through the oral route (89%) at a dose of 4 mg (89%). Of the 12 participants who reached T1, 58% continued at this dose. One participant ceased dexamethasone as there was no perceived benefit. Four patients had the dose reduced without documentation of the reason. None stopped because of an adverse event.

**Table 2.** Dose and Administration Route of Dexamethasone at T0

Initial dosing of dexamethasone at T0			
Dose	***n*** = 18, ***n*** (%)	Administration route	
4 mg once daily	16 (89)	Oral	14
		Subcutaneous	2
2 mg once daily	1 (6)	Oral	1
5 mg twice daily	1 (6)	Oral	1

### Impact on fatigue

At T0, patients' median NCI-CTCAE fatigue scores were 3, signifying their fatigue was not relieved by rest and it limited self-care. Median VAFS scores were 3 and median SAS fatigue scores were 7. At T1 (*n* = 12), improvement was seen in all measures of fatigue although not all were statistically significant. The NCI-CTCAE fatigue scores were reduced to 2.5 (*p* = 0.18). The VAFS scores increased to 5 (*p* = 0.126) and the SAS scores decreased to a median of 5.5 (*p* = 0.007), implying a significant reduction in the distress from fatigue. These results are summarized in Table 3.

**Table 3.** Dexamethasone's Impact on Fatigue at T1

	T0 (***n*** = 18)	T1 (***n*** = 12)	***p***
Median SAS score Patients level of distress from fatigue; 0 = no distress, 10 = worst possible distress	7	5.5	0.007
Median CTCAE fatigue score	Grade 3 Fatigue not relieved by rest; limiting self-care ADLs	Grade 2.5 Fatigue not relieved by rest; limiting instrumental ADLs	0.18
Median VAFS score Patients global fatigue; 0 = worst, 10 = normal	3	5	0.126

ADL, activity of daily living; CTCAE, Common Terminology Criteria for Adverse Events; SAS, symptom assessment scale; VAFS, visual analogue fatigue scale.

### Side effects

Table 4 gives the reported adverse events in the 12 complete data sets. New or worsened reported adverse events at T1 include depression (*n* = 1), delirium (*n* = 1), agitation (*n* = 2), increased appetite or weight gain (*n* = 4), and lower limb edema (*n* = 1). Of these, only one participant experienced a grade 3 adverse event with delirium, the remainder were grade 1 or 2. Depression improved in three patients and insomnia in two patients.

**Table 4.** Reported Adverse Events of Those Participants Who Reached T1, Including Severity

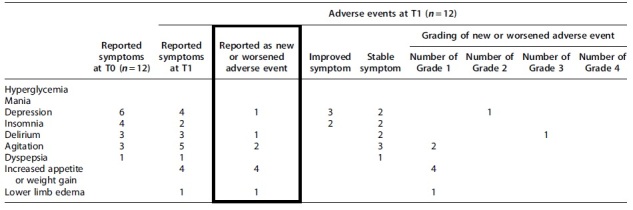

## Discussion

This article suggests that dexamethasone may have a role in managing cancer-related fatigue without causing patients' significant harm. However, only a small number of participants were recruited that may suggest that dexamethasone is not widely used specifically for fatigue management in the region this study was conducted. This is in keeping with current guidelines.^18–20^ It is assumed that instead it is prescribed to target multiple symptoms (such as nausea, anorexia, or pain), with improvement in fatigue being a secondary benefit. Other studies have shown that dexamethasone improves specific symptom clusters such as the fatigue, anorexia–cachexia, and depression cluster.^21^ All participants had a cancer diagnosis, which suggests it is not widely used in the noncancer population.

Our results suggest an improvement in fatigue scores over time from baseline to T1. Similarly, in a randomized controlled trial conducted by Yennurajalingam et al., they found that dexamethasone (*n* = 43) was significantly superior compared with placebo (*n* = 41) for fatigue in patients with advanced cancer.^22^ However, in that trial, they used a higher dose of 4 mg twice daily for 14 days. The average dose reported in this study was 4 mg daily, with the effects monitored for a maximum of seven days. A more recent phase 2 study by Miura et al.^23^ used 8 mg oral or 6.6 mg intravenous dexamethasone daily (days 1–7) followed by 4 mg orally or 3.3 mg intravenous daily (days 8–14).

They failed to validate Yennurajalingam et al.'s^22^ efficacy of dexamethasone in the inpatient setting, but their results did show an improvement in Functional Assessment of Chronic Illness Therapy (FACIT)-fatigue subscale scores compared with baseline at days 8 (*n* = 24) and 15 (*n* = 16).^23^ Further studies are needed to confirm both the potential benefits of dexamethasone for fatigue and the optimal dosing.

Dexamethasone appears to have been well tolerated. One patient had a clinician-reported adverse event of grade 3 delirium, all other new or worsened adverse events were grade 2 or less. This is reassuring, particularly given the potency of dexamethasone. It should be acknowledged that dexamethasone has long pharmacodynamic effects and, therefore, the potential for drug accumulation and increased adverse drug effects.^24^ Given that the study duration was only seven days, the frequency and severity of adverse events may be under-represented.

Neurological or psychiatric adverse events are one of the more commonly reported adverse events associated with corticosteroids as they easily pass into the blood–brain barrier. These effects are most pronounced with dexamethasone due to its high protein binding affinity and longer half-life in cerebrospinal fluid.^24^ It was not possible to determine whether there were other contributing factors to this patient's delirium, and whether the prescribed dexamethasone was the primary cause; reporting of surrogate markers of other confounders, such as C-reactive protein, was poorly completed, and data on comedication were not collected.

There were many positive outcomes with improvements in appetite/weight gain (*n* = 4), depression (*n* = 3), and insomnia (*n* = 2) at T1. The improvement in insomnia, in particular, is surprising; studies suggest that the incidence of sleep disturbance with corticosteroid use is at least 20%.^25^ It is possible that other interventions, such as pre-emptive coprescribing of sleeping medications that were not captured in the study data, are responsible for this improvement and not the dexamethasone.

This study is limited by its observational nature and small sample size that prevented further statistical analysis. The aim was to recruit 100 participants, but after 26 months, the study was closed early as no new data had been obtained in over 6 months despite multiple attempts to increase enrollment. Given the difficulty of recruiting patients, it is unlikely that study numbers could be increased in future similar studies; other studies have had similar issues.^23^ Indeed, in the setting of increasing immunotherapy use, oncologists may have concerns about using concurrent steroids given the potential risk of decreasing the efficacy of immunotherapy.^26,27^

Instead, there may be benefits in a secondary analysis of using dexamethasone for other symptoms or considering a trial where dexamethasone is used for multiple symptoms such as fatigue and anorexia. The short study duration means that it is unclear whether the potentially positive effects are maintained for longer than one week, nor whether there are later side effects such as proximal myopathy. This is particularly important as dexamethasone is generally prescribed for longer than seven days. The documented adverse events are clinician reported so may be over-/under-reported, in particular the impact on sleep and mood. Only advanced cancer patients were recruited and so the results are not representative of the nonmalignant palliative population.

## Conclusion

Dexamethasone is a potential treatment option for cancer-related fatigue. Our results suggest that a once-daily dose of dexamethasone may lead to an improvement in fatigue scores over time, and that dexamethasone is generally well tolerated. The main limitation is the small number of participants who were recruited that may suggest that dexamethasone is not widely used in clinical practice for fatigue alone. Further studies are needed to evaluate its use.

## Abbreviations Used

ADL: activity of daily living
CTCAE: Common Terminology Criteria for Adverse Events
FACIT: Functional Assessment of Chronic Illness Therapy
IMPACCT: Improving Palliative, Aged and Chronic Care through Clinical Research and Translation
IQR: interquartile range
IV: intravenous
NCI-CTCAE: National Cancer Institute Common Terminology Criteria for Adverse Events
RCT: randomized controlled trial
SAS: symptom assessment scale
UTS: University of Technology Sydney
VAFS: visual analogue fatigue scale
